# Automated Quantification of Interlaminar Delaminations in Carbon-Fiber-Reinforced Polymers via High-Resolution Ultrasonic Testing

**DOI:** 10.3390/polym15244691

**Published:** 2023-12-13

**Authors:** Khaled Matalgah, Pruthul Kokkada Ravindranath, Daniel Pulipati, Trevor J. Fleck

**Affiliations:** Department of Mechanical Engineering, Baylor University, One Bear Place, #97356, Waco, TX 76798-7356, USA; khaled_matalgah1@baylor.edu (K.M.); daniel_pulipati@baylor.edu (D.P.)

**Keywords:** CFRP, delamination, ultrasonic testing, non-destructive testing

## Abstract

This article presents a method of ultrasonic testing (UT) that detects and quantifies interlaminar delaminations in CFRP composites with high resolution in terms of both spatial resolution in the planar dimension and depth into the laminate. Unidirectional and woven CFRP laminates were fabricated for this study, with a PTFE film inserted at various depths throughout the laminate to act as intentional crack initiation sites. All samples were mechanically tested via a three-point, end-notched flexure (ENF) test, followed by a quantification of the extent of the induced interlaminar delaminations using UT and X-ray computed tomography (CT). UT analysis for unidirectional CFRP samples was able to show a clear contrast between the delaminated area and the non-delaminated area. UT analysis of the woven CFRP samples yielded comparable results but required finer tuning of analysis parameters due to the interlocking woven fabric. CT results revealed a significant contrast between the crack and composite; thus, fine geometrical features of the crack front could be observed. UT and CT measurements were then compared, revealing an average difference of 1.09% in the delamination area, with UT overestimating as compared to CT. A UT depth study was also performed to automatically locate the interlaminar delamination at different depths throughout the components, with the delamination being predicted within one lamina interface for all samples. These results demonstrate UT’s ability to accurately detect and quantify the extent and location of interlaminar delaminations due to bending.

## 1. Introduction

Carbon-fiber-reinforced polymers (CFRPs) are extensively used in a wide variety of applications, including the aerospace, automotive, and civil engineering industries, among others [1]. Their wide usage in these industries is due to their valuable mechanical properties, which include high strength-to-weight ratios, excellent corrosion resistance, and high fatigue resistance [2]. However, while their properties are advantageous, interlaminar delaminations can be caused by a wide variety of manufacturing defects, such as hole drilling or foreign debris serving as crack initiation sites, as well as in-service defects, such as delaminations caused by impact or excessive deformation. Internal defects or delaminations found in aircraft structures can compromise the structural integrity of aircraft components, potentially leading to catastrophic failures [3]. These delaminations, often hidden from visual inspection, reduce the overall mechanical performance of the structure and are known to accelerate fatigue failure in CFRP components [4]. The literature has quantified this by showing that the compression-after-impact (CAI) strength of CFRP composites is heavily dependent on the delamination area [5,6,7].

Delaminations are difficult to detect and assess due to their sub-surface nature. As such, measuring the delamination area accurately is vital to ensuring the safe use of CFRP components and projecting future in-service performance. Since CFRPs are typically non-homogeneous and anisotropic, detection and evaluation to maintain structural integrity are particularly difficult and require complex analysis and inspection techniques [8].

Non-destructive testing (NDT) is commonly used to evaluate the delamination area, as this non-invasive method can be used without exacerbating the damage and provide insights into the structural integrity of CFRP components [9]. However, as various finite element analysis (FEA) methods advance, high-fidelity NDT measurements are increasingly needed to provide accurate insights into the future viability of CFRP components with interlayer delaminations.

Multiple NDT methodologies, including eddy current testing (ECT) [9,10], infrared (IR) thermography [9,11,12], ultrasonic testing (UT) [9,11,12,13,14,15,16,17], and X-ray computed tomography (CT) [9,13,14,17,18], have advanced to the point where they can provide high-fidelity measurements of CFRP delaminations. Of these prominent NDT methods, CT is considered the most accurate, as it uses high-resolution imaging to visualize internal structures in very accurate detail [19]. Through the use of computational reconstruction, CT creates 3-D images of minute flaws (voids, fractures, pores, etc.) from radiographs taken at a number of different lighting angles. Image contrast is affected by changes in how much the examined object’s X-rays are attenuated [20]. Even though CT is commonly used in the medical, industrial, and material fields [21], CT is comparatively expensive, has size restrictions, is time-consuming, and is limited in terms of deployment in situ [22].

As such, there is a need to further develop versatile and cheaper NDT analysis techniques capable of providing high-fidelity data for FEA predictive analytics. UT is often preferred to CT for certain applications due to its accessibility and portability, its real-time inspection, cost effectiveness, surface accessibility, and the fact that it is able to provide high-resolution images and detect small defects with great accuracy by utilizing high-frequency sound waves [23]. A transducer, operating in a pulse-echo scheme, is used to propagate high-frequency ultrasonic waves through a material, and cracks or delamination found within the material cause the waves to scatter and reflect. These scattered and reflected waves are detected using the same transducer, which is then used to analyze, identify, and characterize interlaminar cracks [24].

Blackman et al. [23] presented a technique to analyze the captured high-resolution C-scan from full-waveform analysis in conjunction with pulse-echo ultrasound for automatic the sizing of foreign objects within carbon-fiber laminates with minimal user input and without prior knowledge of the shape of the defect. A circular polytetrafluoroethylene film was used as the foreign object, placed during manufacturing at different depths. The results showed an average error of 6% of the true area and an absolute error of 0.1 mm.

Castro et al. evaluated and quantified the delamination area in CFRPs’ post -mechanical testing via IR, ECT, UT, and CT. An algorithm of image processing and classification was used for all methods, concluding that ECT and IR were better suited for a qualitative approach, while UT and CT presented good quantitative results [9].

As such, this study focused on developing novel UT data analysis methods to more accurately detect, quantify, and locate interlaminar delaminations due to bending in CFRP components. Given the demonstrated accuracy of X-ray CT, these analysis methods were benchmarked against measurements of an industrial CT machine. To demonstrate the flexibility of the developed analysis techniques, a variety of laminated CFRP samples were considered, all of which were fabricated with intentional crack initiation sites at different depths. All samples were mechanically tested via a three-point bend test to induce interlaminar delaminations. These delaminations were scanned using both UT and CT techniques. UT data analysis quantified the delamination area, spatial location, and depth, which were then plotted against the obtained CT measurements. The novelty of this study is that it assessed the accuracy of a UT data collection and analysis approach compared to CT, in an attempt to develop an efficient, inexpensive approach to identifying critical defects with high resolution.

## 2. Materials and Methods

### 2.1. Specimen Fabrication

**Figure 1:**
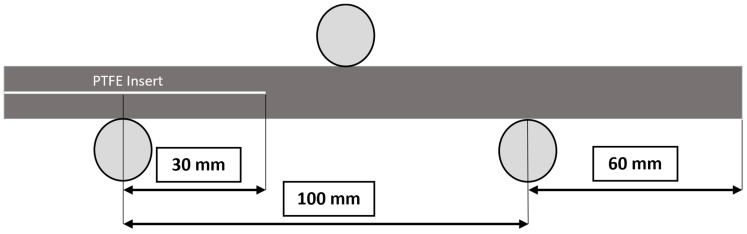
Three-point bend ENF testing configuration.

In this study, to demonstrate the flexibility of the developed UT measurement and analysis methods, four different carbon-fiber-laminated composite panels were manufactured. The first panel was fabricated using a 3K, 6 oz., plain-weave carbon fiber using the vacuum-assisted resin molding (VARTM) technique, composed entirely of 18 (0°) laminas with Proset INF 114 resin and Proset INF 211 hardener (Pro-Set Inc., Bay City, MI, USA). The other three panels were fabricated using unidirectional prepreg (carbon fiber + 250F resin system) manufactured using a hot press (Carver, 3893L4PLI006, Wabash, IN, USA), under the manufacturer’s recommended temperature cycle and at a constant pressure of 275 kPa, and composed entirely of 30 (0°) laminas. During manufacturing, a polytetrafluoroethylene (PTFE) film of 50 µm thickness (McMaster-Carr, SF103V0002, Los Angeles, CA, USA) was inserted in the midplane of the woven panel and in the first unidirectional prepreg panel. The PTFE film was also inserted at approximately one-third of the thickness from the surface (between laminas 8 and 9) into the second unidirectional panel and at approximately two-thirds of the thickness from the surface (between laminas 21 and 22) into the third unidirectional panel. The PTFE film acted as an intentional initiation site for interlaminar cracks under mode-II loading conditions, with the aforementioned locations providing delaminations at various depths into the components. Delaminations were initiated at the midplane for woven and unidirectional panels in order to verify the ability to locate delaminations in multiple material sets. All four panels were then cut via a tile saw into coupons (3 from each panel) of dimensions 180 × 25 mm to follow the ASTM standard D7905, as shown in Figure 1 [25].

### 2.2. Fracture Test

Three-point bend tests were conducted using a Universal Test Machine (SM-1000-294, Test Resources, Shakopee, MN, USA) with a loading capacity of 4.4 kN. The test was set up following the ASTM standard D7905 [25], with a representative ENF sample, shown in Figure 1. The test machine was operated in displacement control mode with a constant displacement rate of 1 mm/min. Due to unstable delamination growth in three-point bend quasi-static mode-II tests [25], the test was stopped for each sample once the critical displacement was reached and unstable crack growth occurred at the PTFE location.

### 2.3. Ultrasonic Scanning Set Up

**Figure 2:**
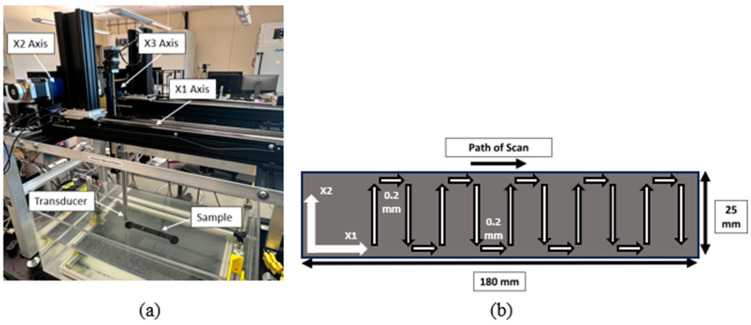
Ultrasonic testing set up with (**a**) custom UT immersion system and (**b**) representative rastering pattern (0.2 mm spacing used in all studies).

Ultrasonic C-scans were performed on the tool side of the woven ENF sample after three-point bend mechanical testing had occurred. A custom-built, pulse-echo C-scan immersion system was used for all UT scans in conjunction with an Olympus Focus PX Flaw Detection system, as shown in Figure 2a. For this study, a 10 MHz, 38.1 mm (1.5”) nominal focal length, spherically focused transducer (A311S-SU-F1.5N-PTF, Olympus Corporation, Tokyo, Japan) was used. The transducer was focused at 50% depth into the sample. During each scan, the transducer followed a raster pattern, as depicted in Figure 2b, with 0.2 mm raster resolution in both the *X*_1_ and *X*_2_ directions, with *X*_1_ aligning along the length of the sample and *X*_2_ aligning transversely to *X*_1_, as shown in Figure 2a. After each UT scan was completed, a custom MATLAB script was used to analyze the data to automatically identify the depth of the crack, as well as quantify the spatial location and geometry of the crack front, which is further discussed in Section 3.1 and Section 3.2.

### 2.4. X-ray CT Scanning and Analysis

**Figure 3:**
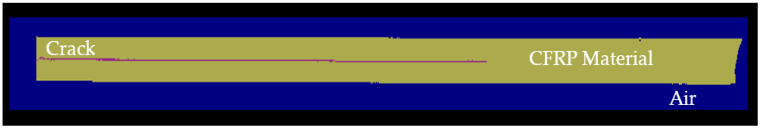
User-painted frame showcasing three different features: crack (pink), material (yellow), and air (blue).

**Figure 4:**
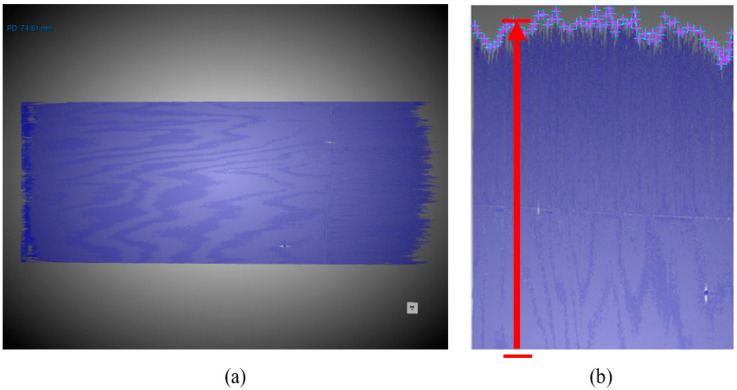
CT-analyzed image of a crack with (**a**) isolated crack volume and (**b**) representative crack length.

CT scans were performed using a North Star Imaging (NSI) X-3000 microfocus X-ray system (Rogers, MN, USA). To achieve the necessary scans for the analysis, a voltage of 80 kV and current of 850 µA, with a focal spot size of 68 µm, was used to generate an X-ray beam with a voxel size of 29.21 µm. NSI’s in-house reconstruction software (efX-CT 2.2.4.0) was used to perform the reconstruction after the scan was complete, by selecting the sharpness and beam hardening settings for each individual scan. The data were analyzed using Dragonfly software’s Segmentation Wizard feature, which uses artificial intelligence (AI). The AI feature of this software was trained to differentiate between the crack, the CFRP material, and the background (air) based on the difference in the grayscale values from the CT scan, as shown in Figure 3. Once the AI feature was trained, the software was able to detect similar grayscale values through all the slices of the scanned sample, and segmentation was performed. The crack throughout the sample was separated from the rest of the material, and pores/voids were also separated from the segmented crack, as shown in Figure 4a. A slice image of this separated crack region of interest (ROI) was imported into MATLAB (R2023a), where the *getpts* function was used for further analysis [26]. The function first prompts the user to select points of the bottom edge of the crack to act as the reference point of origin and then prompts the user to select multiple points of the crack front. Each point is chosen to capture the essential characteristics of the crack geometry. The calculation measurement of the crack front involves measuring the distance between the bottom edge of the crack and crack front. The red arrow in Figure 4b shows how a representative measurement of an individual location was obtained.

## 3. Analysis

An in-house MATLAB script was developed to automatically identify the depth of the delamination and accurately quantify the geometry and spatial location of the delamination crack front. The analysis calculated the output of the dimensions of the crack in the *X*_1_/*X*_2_ plane and where the predicted lamina interface was located. This was achieved via analysis of the full waveform (A-scan) at each data collection point, as described in Section 3.1 and Section 3.2. Prior to the data analysis developed in this work, the raw data obtained from the Olympus Focus PX were converted to MATLAB format, smoothed, and aligned, as per previous work [23,27]. Once scanning was complete, the data were saved in .fpd format, which was subsequently converted to and saved as a 3-dimensional matrix via MATLAB, in which the A-scans for each location were stored for the (*X*_1_,*X*_2_) location. Prior to this analysis, the individual A-scans collected in increments of 0.2 mm in both the *X*_1_ and *X*_2_ location were smoothed using a Gaussian smoothing technique in which the transducer signal was averaged across its nearest neighbors to reduce noise [23]. Next, each A-scan was shifted relative to the front wall using a detection threshold, aligning these signals in time to ensure consistent depths from one spatial location to the next, maintaining uniformity in the *X*_1_/*X*_2_ plane throughout the depth of the component. Once the data were smoothed and aligned, the depth of the delamination was identified, and the resultant crack was measured at the identified *X*_1_/*X*_2_ plane, as per Section 3.1 and Section 3.2.

### 3.1. Delamination Depth Identification through UT Signal Analysis

**Figure 5:**
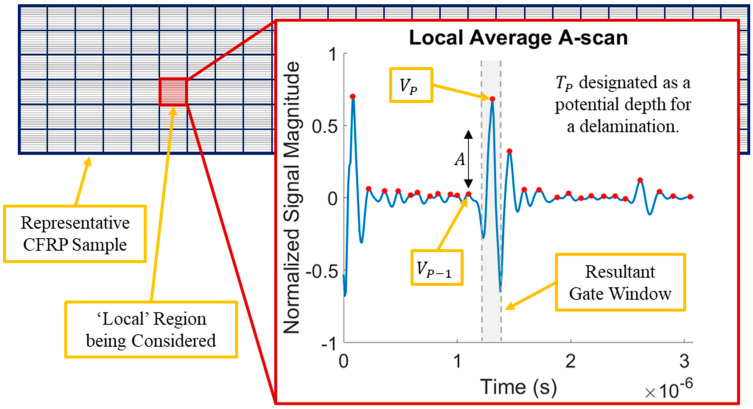
Depiction of CFRP sample divided into local regions, with a representative local average A-scan of a unidirectional sample.

Once the data were smoothed and aligned, they were sectioned into 2 mm × 2 mm “local” regions in the *X*_1_/*X*_2_ pane, as depicted in Figure 5. The average A-scan was calculated for each region and the local maxima of each regional average A-scan were identified. These local maxima have been shown to correspond to individual lamina in CFRP laminates [23,27]. It should be noted that these reflections were less pronounced in the unidirectional samples than in the woven samples, with a representative A-scan of a unidirectional sample shown in Figure 5. These local maxima (V_p_) were then considered with respect to the previous local maxima (V_p-1_) in time. Each local maximum (or lamina interface reflection) that was greater by a specified amplitude than the previous local maximum, depicted as A in Figure 5, was flagged as a potential delamination location. This approach was considered because the signal for non-delaminated samples attenuated uniformly throughout the depth of the sample with minimal increases in the lamina interface reflections. This approach flagged any delaminations in a local region significant enough to result in a reflection at that location. After automated processing of each local average A-scan, the depth that contained the most flags for potential delaminations was chosen as the depth of interest moving forward in this study. It should be noted that a normalized response value of 0.3 for A was used for the samples herein, which was based on the individual materials and corresponding signal responses considered.

### 3.2. Quantification of Delamination Geometry and Spatial Location

**Figure 6:**
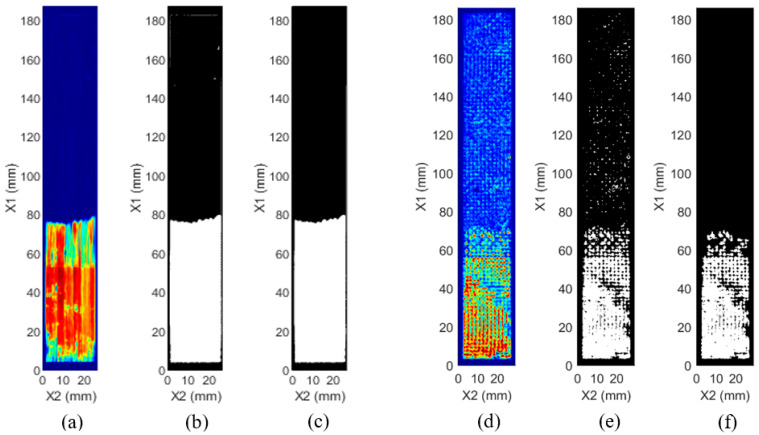
Ultrasonic testing results with (**a**,**d**) traditional C-scan images and (**b**,**e**) threshold images with (**c**,**f**) small artifacts filtered out for unidirectional and woven samples, respectively.

At the depth of interest obtained from the analysis in Section 3.1, a gated C-scan was visualized by creating a window in time around the depth of interest, as represented pictorially by the dashed gray lines shown in Figure 5. For the samples herein, a gate of 30 data points was used, with respect to the identified depth of interest. When considering the sampling rate used (100 MHz), this corresponds to a time gate ($t_{g}$) of 0.3 µs. For each individual A-scan, a proxy for the energy of the signal response within this gate, $E(X_{1},X_{2})$, was calculated for each spatial location (*X*_1_, *X*_2_) as:

$$E(X_{1},X_{2})=\int_{t_{p}-\frac{t_{g}}{2}}^{t_{p}+\frac{t_{g}}{2}}V{\left(t,X_{1},X_{2}\right)}^{2}dt$$

in which $E(x_{1},x_{2})$ represents the value of each pixel shown in Figure 6a,d; $t_{p}$ is the time of the peak in signal response corresponding to the identified local maxima from 3.1; $t_{g}$ is the time width of the gate (0.3 µs); and $V(t,X_{1},X_{2})$ is the signal response (or A-scan) at each (*X*_1_, *X*_2_) location. This resulted in a gated C-scan image, as shown in Figure 6a for a representative unidirectional sample and Figure 6d for a representative woven sample. The C-scan image was analyzed, and the regions with and without the crack were differentiated to black and white based on a threshold, as shown in Figure 6b,e. The value of the threshold was based on the regional average A-scan signals without any delamination indicators, or the “clean” local average A-scans from Section 3.1. For the “clean” regions, the average and standard deviation of the energies from each “clean” scan location were calculated to statistically represent the variations seen in the clean signal response. The locations with a calculated energy outside a specified number of standard deviations (20 for the samples herein) from the clean response were then designated as locations with delamination (white). Any energy within the specified number of standard deviations was considered clean (black). The resultant image was then filtered to create Figure 6c for a representative unidirectional sample and Figure 6f for a representative woven sample, to remove any small amounts of damage known to be below the delamination area of concern. For the samples herein, groupings of pixels below 80 pixels and effective delamination sizes smaller than 3.2 mm^2^ were removed from consideration. It should be noted that while clusters of 80 pixels were removed across all samples, this filtering was primarily necessary for the woven samples due to the higher variance in signal response, which is further discussed in Section 4.2. The full time taken to analyze the data from each sample was approximately 35 s, progressing from the 3-dimensional matrix of A-scan data to outputting the geometry and depth of the delamination (example shown in Figure 6c,f).

## 4. Results and Discussion

### 4.1. Ultrasonic Testing Results and Discussion

Representative ultrasonic C-scan images, along with subsequent image processing steps, are shown in Figure 6, including both unidirectional (Figure 6a–c) and woven samples (Figure 6d–f). Figure 6a shows a clear contrast between the damaged area (red) and undamaged area (blue) for the unidirectional samples. The same analysis of the woven samples, as shown in Figure 6d, did not yield the same clarity. This highlights the need for further image analysis to assist with delamination quantification of woven CFRPs. Figure 6b,e, showing unidirectional and woven samples, respectively, display the binary C-scan images, as described in Section 3.2. These binary images were filtered, removing artifacts as shown in the figure (Figure 6c,f) to determine the delamination size, as discussed in Section 3.2.

The obtained depth prediction results from the developed analysis, as reported in Table 1, demonstrate the ability to accurately locate the delamination in terms of depth into the component. The results indicate that the algorithm described previously is able to effectively detect the delamination depth within the laminate, as the “depth of interest” discussed in Section 3.1, which aligns with the actual laminate structure, with all measurements being within one lamina interface of the actual delamination.

### 4.2. Comparison of UT and X-ray CT results

**Figure 7:**
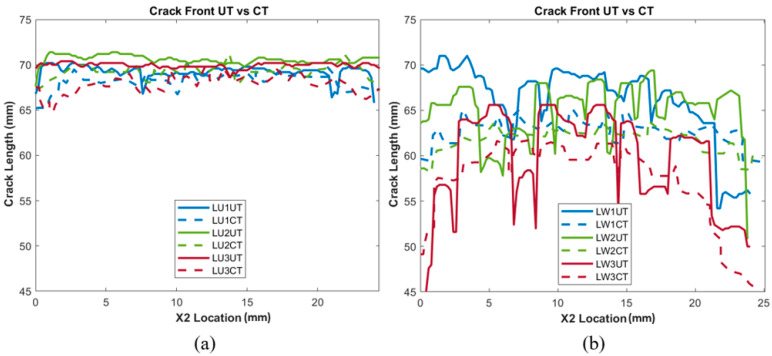
Crack length vs. *X*_2_ direction for all (**a**) unidirectional prepreg (1–3) and (**b**) woven VARTM samples.

**Figure 8:**
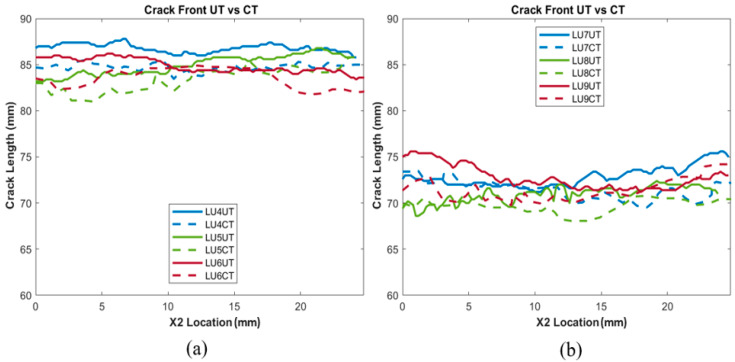
Crack length vs. *X*_2_ direction for all (**a**) unidirectional prepreg (4–6) and (**b**) unidirectional prepreg (7–9) samples.

The delaminated crack areas calculated from the data collected from the UT scans, as well as CT scans, are tabulated in Table 2 and Table 3 for comparison purposes. The average perfect difference amongst all the unidirectional samples was 1.05%, and the average percent difference amongst the woven samples was 1.21%, indicating that the developed UT analysis showed accuracy when measuring unidirectional and woven samples, compared to a CT scan baseline. Figure 7 and Figure 8 show all of the crack front measurements plotted as a function of *X*_1_ location, giving insights into how accurately the UT can position the crack front in the *X*_1_ and *X*_2_ plane. However, for woven crack front measurements, shown in Figure 7b, it should be noted that greater fluctuations and variability were observed than in the CT scan baseline. A difference in length in the X_2_ direction (i.e., along the width of the sample) between UT and CT of the same sample was observed. This could be caused by a variety of reasons. The most likely reason is the scattering caused by the overlapping and undulating fiber tows, which cause greater variation in the signal response across the woven sample. Ultrasound waves undergo various interactions at the edges of materials and interfaces, leading to reflections and diffractions of the wave that impact the reliability and accuracy of measurements at the edges [28]. A greater variance in signal response would have resulted in difficulty in distinguishing between damage and the natural reflections and scattering of the woven material. This is displayed when comparing Figure 6b,e, in which the unfiltered, binarized image shows a clear difference in response. Interlaced sets of fibers at right angles, which lead to the ultrasonic waves experiencing complex interactions and reflections, can be observed, supporting the above hypothesis.

In order to validate the consistency and reliability of the UT measurement technique at varying depths, samples in Table 3 were scanned on the alternate side and the delaminated crack areas were measured and compared, as tabulated in Table 4. The average percent difference amongst the samples was 0.77%, indicating the accuracy of the UT analysis, regardless of depth, for the samples considered.

## 5. Conclusions

An ultrasonic testing analysis technique to measure and locate interlaminar delaminations in carbon-fiber-reinforced polymer composite laminates was developed. The results were benchmarked against X-ray computed tomography results, in which the delaminated area was measured within 1.05% and 1.21% difference for unidirectional and woven laminates, respectively. Plots of the crack length as a function of the width of the sample showed not only accuracy in terms of the calculated area but consistency along the length of the crack, locating the geometry of the crack in the *X*_1_/*X*_2_ plane. These findings show that the UT method developed in this study can precisely identify and determine the extent of interlaminar delaminations due to bending and thus act as a reliable alternative to CT while maintaining advantages in terms of cost and time. In addition to accurately characterizing the geometry and location of the delamination in the *X*_1_/*X*_2_ plane, automated analysis determined the depth of each of the delaminations identified in this study within one lamina interface. The high fidelity achieved in this analysis would allow for the precise location of damaged and delaminated regions, which can be used in subsequent FEA predictive analytics studies.

## Figures and Tables

**Table 1:** Comparison of PTFE insert’s actual vs. predicted position.

	PTFE Actual Position	PTFE Predicted Position	Lamina Inaccuracy
LU1	Between 15 and 16 Lamina	Between 15 and 16 Lamina	0
LU2	Between 15 and 16 Lamina	Between 14 and 15 Lamina	1
LU3	Between 15 and 16 Lamina	Between 14 and 15 Lamina	1
LU4	Between 21 and 22 Lamina	Between 21 and 22 Lamina	0
LU5	Between 21 and 22 Lamina	Between 21 and 22 Lamina	0
LU6	Between 21 and 22 Lamina	Between 21 and 22 Lamina	0
LU7	Between 8 and 9 Lamina	Between 9 and 10 Lamina	1
LU8	Between 8 and 9 Lamina	Between 9 and 10 Lamina	1
LU9	Between 8 and 9 Lamina	Between 8 and 9 Lamina	0

**Table 2:** Comparison of crack area measurements made using UT and CT.

NDT Method	Crack Area Measurement (mm^2^)					
	LU1	LU2	LU3	LW1	LW2	LW3
UT	1662.1	1751.0	1706.8	1589.2	1552.2	1433.4
CT	1693.0	1721.2	1690.8	1555.2	1536.0	1427.9
% Difference	1.83	1.73	0.95	2.19	1.05	0.39

**Table 3:** Comparison of crack area measurements made using UT and CT.

NDT Method	Crack Area Measurement (mm^2^)					
	LU4	LU5	LU6	LU7	LU8	LU9
UT	2083.9	2053.6	2100.4	1793.1	1742.2	1787.3
CT	2100.7	2039.1	2105.5	1791.4	1688.5	1789.4
% Difference	0.81	0.71	0.24	0.01	3.08	0.12

**Table 4:** Comparison of crack area measurements by UT of both sides.

NDT Method	Crack Area Measurement (mm^2^)					
	LU4	LU5	LU6	LU7	LU8	LU9
UT	2083.9	2053.6	2100.4	1793.1	1742.2	1787.3
UT-Flip	2052.3	2043.0	2097.2	1780.1	1733.8	1765.4
% Difference	1.52	0.52	0.15	0.73	0.48	1.23

## Data Availability

Data will be provided upon request to the corresponding author.
